# Conserved Mitotic Phosphorylation of a Proteasome Subunit Regulates Cell Proliferation

**DOI:** 10.3390/cells10113075

**Published:** 2021-11-08

**Authors:** Jinyuan Duan, Wenzhu Li, Xin Shu, Bing Yang, Xiangwei He, Xing Guo

**Affiliations:** 1 Life Sciences Institute, Zhejiang University, Hangzhou 310058, China; jyduan@zju.edu.cn (J.D.); wenzhuli@zju.edu.cn (W.L.); sx725@zju.edu.cn (X.S.); bingyang@zju.edu.cn (B.Y.); xhe@zju.edu.cn (X.H.); 2 Zhejiang Provincial Key Laboratory for Cancer Molecular Cell Biology, Hangzhou 310058, China

**Keywords:** cell cycle, mitosis, proteasome, PSMA5/α5, phosphorylation

## Abstract

Reversible phosphorylation has emerged as an important mechanism for regulating proteasome function in various physiological processes. Essentially all proteasome phosphorylations characterized thus far occur on proteasome holoenzyme or subcomplexes to regulate substrate degradation. Here, we report a highly conserved phosphorylation that only exists on the unassembled α5 subunit of the proteasome. The modified residue, α5-Ser16, is within a SP motif typically recognized by cyclin-dependent kinases (CDKs). Using a phospho-specific antibody generated against this site, we found that α5-S16 phosphorylation is mitosis-specific in both yeast and mammalian cells. Blocking this site with a S16A mutation caused growth defect and G2/M arrest of the cell cycle. α5-S16 phosphorylation depends on CDK1 activity and is highly abundant in some but not all mitotic cells. Immunoprecipitation and mass spectrometry (IP-MS) studies identified numerous proteins that could interact with phosphorylated α5, including PLK1, a key regulator of mitosis. α5–PLK1 interaction increased upon mitosis and could be facilitated by S16 phosphorylation. CDK1 activation downstream of PLK1 activity was delayed in S16A mutant cells, suggesting an important role of α5-S16 phosphorylation in regulating PLK1 and mitosis. These data have revealed an unappreciated function of “exo-proteasome” phosphorylation of a proteasome subunit and may bring new insights to our understanding of cell cycle control.

## 1. Introduction

The cell cycle is essential for all cellular life and is tightly regulated throughout the entire living history of an organism [1]. Dysregulation of this process underlies multiple diseases such as cancer [2,3]. A complete cell cycle consists of G1, S, G2, and M phases. Different cyclin proteins pair up with their cognate cyclin-dependent kinases (CDKs) at different cell cycle stages, forming a major driving force of cell cycle progression. The M phase (mitosis), although taking up only a small fraction of the cell cycle, is accompanied with the most drastic changes of the cell, which lead to the ultimate birth of two daughter cells. A master regulator of mitosis is cyclin B/CDK1 (also known as cdc2), which phosphorylates a myriad of proteins involved in all relevant aspects of the cell, including DNA condensation, spindle/kinetochore formation, membrane/organelle reorganization, and sister chromatid separation [4,5]. Several other kinases (e.g. PLK1, Aurora kinases) and phosphatases (e.g. PP1, PP2A, and Cdc25s) also play critical roles in the phospho-regulation of mitosis [4]. Another quintessential component of the vast signaling network of cell cycle control is the ubiquitin–proteasome system (UPS), which degrades almost all cell cycle-related proteins in a highly regulated manner with extraordinary spatiotemporal preciseness [6]. By irreversibly eliminating these proteins, the UPS ensures that the cell cycle can only move in one direction.

At the central hub of the UPS is the 26S proteasome, which is responsible for degrading the vast majority of cellular proteins [7]. In a fully assembled 26S proteasome, one or two 19S regulatory particles (RPs) cap one or both ends of the 20S core particle (CP). Ubiquitinated protein substrates are captured and unfolded by the 19S RP then fed to the 20S CP for proteolysis. The RP contains 6 AAA^+^-type ATPases (Rpt1–6) and 13 non-ATPase subunits (Rpn1–3, 5–13, and 15), with Rpt1-6 forming a hexameric ring that directly locks onto the 20S. Two types of structurally similar subunits (α and β) make up the 20S CP. Specifically, α1–7, encoded by different genes, assemble into an α ring, while the seven β subunits (β1–7) form a β ring. Two sets of each ring stack on each other in a α-β-β-α order, giving rise to the cylindrical structure of 20S. Three subunits of the inner (β) rings, β1, 2, and 5, harbor peptidase activities that are enclosed inside the 20S chamber, while the highly conserved N-termini of the α subunits of the outer rings intercalate with each other to serve as a "gate” that prevents undesired entry of a protein. Binding of Rpt1–6 to the α ring in the presence of ATP opens the 20S gate, allowing for substrate access to the catalytic sites. The stepwise assembly pathways of both RP and CP require a number of chaperones [8,9,10]. Although most proteasome subunits are found in the fully assembled 26S holoenzymes, some subunits (e.g., α5) are also present in their free form or in assembly intermediates [11,12,13,14]. Therefore, in cells, there is a dynamic equilibrium among proteasome (sub)complexes of different compositions.

Despite the fundamental importance of the proteasome in almost every cellular activity, nonetheless little is known about whether/how the proteasome itself is regulated under different physiological or pathological conditions. Our previous work has demonstrated unique functions of reversible phosphorylation in regulating proteasome assembly and activity [15,16,17,18,19]. In particular, cell cycle-dependent phosphorylation of Rpt3-Thr25 by the kinase DYRK2 controls proteasome function, cell proliferation, and tumorigenesis, providing the first evidence that the proteasome as a well-established cell cycle regulator is also regulated by cell cycle [17,20,21,22]. A considerable number of proteasome phosphosites are within the SP/TP motif typically recognized by CDKs [16,23]. However, of over 450 phospho-sites found on the human 26S proteasome, only a handful have been functionally characterized, and none of them are CDK targets [16,24]. 

In this study, we investigated the function and regulation of a highly conserved SP site on the 20S subunit α5 that is phosphorylated in yeast and mammalian cells. This site, α5-Ser16, is only phosphorylated in mitotic cells, probably by CDK1/cyclin B. Blocking this phosphorylation led to reduced cell proliferation. To our surprise, S16 phosphorylation occurred only on unassembled α5 outside the proteasome, suggesting a degradation-independent function.

## 2. Materials and Methods

### 2.1. Cell Culture, Transfection, and Infection

293T, MCF7, ZR-75-1, H1975, and H3255 cells were originally purchased from American Type Culture Collection (ATCC). 293A and Neuro2A cells were kindly provided by Dr. Kun-Liang Guan (University of California, San Diego, CA, USA) and Dr. Zhiping Wang (Zhejiang University, Hangzhou, China), respectively. All cells were cultured in DMEM with 10% fetal bovine serum (FBS) and penicillin/streptomycin (ThermoFisher, Waltham, MA, USA), except for the two lung cancer cell lines (H1975 and H3255), which were maintained in RPMI with FBS and Pen/Strep. To keep cells from mycoplasma contamination, we added ciprofloxacin (Sigma, St. Louis, MO, USA) intermittently. Transient transfection was conducted with polyethylenimine (PEI, Polysciences, Warrington, PA, USA). For stable overexpression of proteasome subunits, 293T cells were co-transfected with the pQCXIP-TBHA retroviral vector [17] containing desired cDNAs and the pCL10A1 packaging vector for retrovirus production. Viral media from 293T were passed through a 0.45 µM filter (Millipore, Burlington, MA, USA), mixed with 10 µg/mL polybrene (Sigma), and added to recipient cells. After reaching confluency, infected cells were passaged and selected with puromycin (ThermoFisher, 1 µg/mL). For transient knockdown of α5, the pLKO.1 vector containing a control or an α5-specific shRNA was co-transfected with the psPAX2 and pMD2.G packaging plasmids into 293T cells. Viruses were harvested and used for infection as above, and the target cells were analyzed 48 h post-infection without drug selection.

### 2.2. Plasmids

Plasmids encoding α5-Flag and all the other proteasome subunits were generously provided by Dr. Shigeo Murata (The University of Tokyo, Tokyo, Japan) and subcloned into the pQCXIP-TBHA vector by PCR. Several shRNAs targeting α5 were cloned into the pLKO.1 lentiviral vector and tested. The one shRNA with intermediate knockdown efficiency was chosen, and lentiviruses were produced as described above. Mutagenesis was carried out using the QuikChange™ method (Agilent, Santa Clara, CA, USA). All plasmids were verified by sequencing, and all cloning primers are listed in Table S1. HA-PLK1 was kindly offered by Dr. Fangwei Wang (Zhejiang University, Hangzhou, China). 

### 2.3. Antibodies and Reagents

The anti-α5 (pS16) phospho-specific antibody was generated by immunizing rabbits with the following antigen peptide: Ac-NTF[pS]PEGRLC-NH_2_. IgGs from the antisera were isolated and purified by an antigen-conjugated affinity column. All commercial antibodies used in this paper are listed in Table S2. The fluorogenic peptide substrate Suc-LLVY-AMC was purchased from UBPBio (Dallas, TX, USA). CDK1 inhibitors were purchased from SelleckChem (Houston, TX, USA). Aphidicolin was bought from Millipore. Nocodazole and ATP were bought from Sigma. Calyculin A was from Tocris (Minneapolis, MN, USA).

### 2.4. Cell Proliferation Assay and Flow Cytometry

Cell proliferation was measured by the MTS assay. Equal number of cells were seeded in triplicate in a 96-well plate. At the indicated time, MTS reagent from the CellTiter 96^®^ AQueous One Solution Cell Proliferation Assay kit (Promega, Madison, WI, USA) was added to each well according to the manufacturer’s instruction. After incubation at 37 °C for 30–60 min, Abs490 was measured by a multi-well plate reader (TECAN, Männedorf, Switzerland).

Cell cycle distribution was monitored by propidium iodide (PI) staining as previously described [17]. Cells were analyzed on a FACSCalibur™ flow cytometer (BD Life Sciences, Franklin Lakes, NJ, USA), and data analysis was conducted using the Flowjo software (v.10, BD Life Sciences).

### 2.5. CRISPR/Cas9-Mediated Genome Editing

For generating α5-S16A knock-in in human cells, we designed several guide RNA (gRNA) sequences targeting the surrounding genomic region using the Chopchop website (Available online: http://chopchop.cbu.uib.no/, accessed on 30 August 2021). The one gRNA with the highest editing efficiency (as determined by the SURVEYOR assay) was cloned into the PX458 vector (Addgene) and sequence-verified. For repair donor preparation, genomic DNA from 293T cells was used as template for amplifying the left and right homology arms flanking the Cas9 cleavage site, which is close to the codon of α5-S16 (TCT). Successful knock-in was confirmed by genomic DNA sequencing of the entire region including homology arms and by Western blot with the anti-pS16 antibody. All relevant primer sequences are listed in Table S1.

### 2.6. Immunoblot, Immunoprecipitation, and Streptavidin Pulldown

Cells were lysed with TBSN buffer (50 mM Tris (pH 7.5), 125 mM NaCl, 0.5% NP-40, protease inhibitors, phosphatase inhibitors, 1 mM ATP, 5 mM MgCl_2_). Cleared cell lysates were quantified using Bradford Protein Assay (ThermoFisher). For immunoprecipitation, 2–4 µg of the indicated antibodies was mixed with 0.5–1.0 mg of lysate, and the mixture was incubated at 4 °C for 1 h before the addition of 10 µL Protein G beads (ThermoFisher). After incubation, for another 45–60 min, beads were collected by centrifugation, thoroughly washed with TBS buffer, and boiled in 1× Laemmli sample buffer. Streptavidin pulldown was similarly performed using 8–10 µL High Capacity Streptavidin Agarose (ThermoFisher). Denatured samples were separated on SDS-PAGE and transferred to nitrocellulose membranes for immunoblotting following standard procedures.

### 2.7. Fluorescence Microscopy

Cells grown on coverslips were fixed, permeabilized, and immunostained according to standard procedures. DAPI (Yeasen, Shanghai, China) was added for nuclear staining. Confocal images were taken with a LSM880 Airyscan microscope (Zeiss, Oberkochen, Germany) using a 40× or 60× oil lens. GFP images of *S. pombe* were acquired by a DeltaVision ELITE microscope (GE, Chicago, IL, USA) with a 60× Objective oil lens. 

### 2.8. Yeast Genetics

Pup2-myc strains were constructed by homologous recombination using DNA fragments generated by standard PCR-based amplification. Pup2 (S16A) mutation was constructed by site-directed overlapping PCR. Standard LiOAc-based protocols were used for yeast transformations with plasmids and PCR products. Yeast strains with various combinations of mutations were constructed by genetic crossing and tetrad dissection. 

### 2.9. Sucrose Gradient Ultracentrifugation

Sucrose gradient was prepared in a 5 mL polypropylene centrifuge tube (Beckman Coulter, Brea, CA, USA) using a roller pump (Leadfluid, Baoding, China). Five hundred microliters of cell lysate was gently added to the sucrose solution, and the mixture was ultra-centrifuged at 268,000× *g* for 3 h. After centrifugation, each sample was divided into 200 µL × 24 fractions, from which 20 µL was withdrawn for Western blot analysis.

### 2.10. Mass Spectrometry

Four 15 cm dishes of 293T were grown to 60–70% confluency. Two dishes of 293T were treated with aphidicolin (10 µM) for 12 h, and two dishes of 293T were treated with nocodazole (100 ng/mL) for 16 h. Cells were lysed with lysis buffer (50 mM Tris-HCl, 0.5% NP-40, protease inhibitors, phosphatase inhibitors, 1 mM ATP, 5 mM MgCl_2_). Cell lysate was immunoprecipitated with anti-α5-pS16 and Protein G agarose (ThermoFisher) at 4 °C for 1 h. The precipitated samples were thoroughly washed with TBS buffer and boiled in 1× SDS sample buffer at 95 °C for 10 min. 

One 10-well, 10% SDS-PAGE gel was prepared. Samples were loaded and slightly separated by a short SDS-PAGE run. The gel was stained with Coomassie Blue. After staining, the stained gel lanes were sliced into two pieces, and the gel slices were processed for in-gel tryptic digestion. The proteins in the gel were reduced with 10 mM DTT for 1 h at 56 °C, modified with 55 mM iodoacetamide in 50 mM ammonium bicarbonate in the dark for 45 min at room temperature, and digested overnight with modified trypsin (Promega) in 50 mM ammonium bicarbonate at a 1:10 enzyme-to-substrate ratio at 37 °C. The resulting tryptic peptides from the liquid phase were analyzed by LC–MS/MS using an QExactive HF-X mass spectrometry (ThermoFisher Scientific). The peptides were loaded in solvent A (0.1% formic acid in water) onto a C18 column (75 μm × 15 cm, 1.9 μm C18, 5 μm tip). The peptide mixture was resolved using a (5 to 35%) linear gradient of solvent B (80% acetonitrile with 0.1% formic acid) for 48 min, 35–100% B in 5 min, followed by 100% solvent B for 5 min, using a flow rate of 0.3 μL/min. Mass spectrometry was performed in a positive mode (*m*/*z* 350–1500, resolution 60,000) using repetitively full MS scan (measured in the Orbitrap detector) followed by HCD.

### 2.11. Data Analysis and Presentation

Statistical analyses were conducted with Prism 7 (GraphPad, San Diego, CA, USA) and Excel 2019 (Microsoft, Redmond, WA, USA). Quantification of Western blot results and processing of confocal photographs were performed with ImageJ (v.1.50b, National Institute of Health, Bethesda, MD, USA). Sequence alignment was conducted using MEGA (v.10.0.5, available online: https://www.megasoftware.net/, accessed on 30 August 2021). Mass spectrometry results were searched by MaxQuant 1.6.0.1 (Max Planck Institute of Biochemistry, Planegg, Germany). Protein–protein interaction network and GO term analysis were generated by STRING (available online: https://string-db.org/, accessed on 30 August 2021). Structural models were prepared with PyMol (2.2.0, Schrodinger LLC., New York, NY, USA).

## 3. Results

### 3.1. α5-S16 Is a Conserved Phosphosite Associated with Mitosis

**Figure 1:**
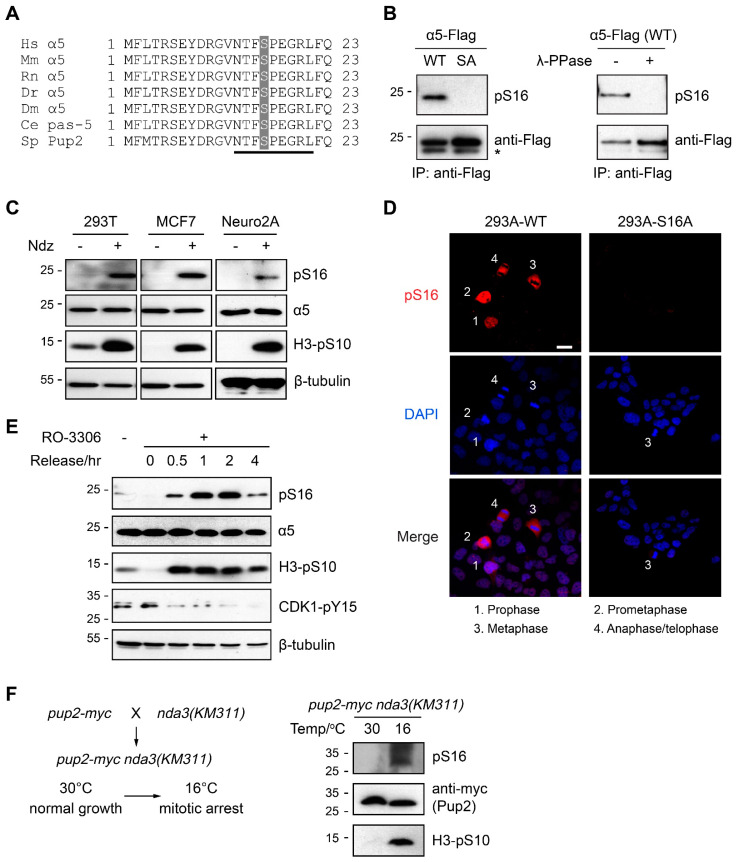
α5-S16 is a conserved mitotic phosphosite. (**A**) Alignment of α5 N-terminal sequences. Ser16 is shaded. The underlined sequence, which is conserved throughout evolution, denotes the antigen peptide used for generating the anti-pS16 antibody. α5 sequences used for alignment are NP_002781 (*H. sapiens*), NP_036097 (*M. musculus*), NP_058978 (*R. norvegicus*), NP_991271 (*D. rerio*), NP_725669 (*D. melanogaster*), NP_492765 (*C. elegans*), and NP_594372 (*S. pombe*). (**B**) 293T cells were transfected with the indicated α5-Flag plasmids and pre-treated with Calyculin A (25 nM, 30 min, to enhance global phosphorylation). Cell lysates were incubated with anti-Flag antibody, and immunoprecipitates were treated with or without λ-phosphatase before Western blot analysis using the anti-pS16 antibody. *, IgG light chain. (**C**) 293T, MCF7, and Neuro2A cells were treated with DMSO or 100 ng/ml of nocodazole (Ndz) for 16 h. Total cell extracts were analyzed by Western blot. H3-pSer10 was used as a mitotic marker. (**D**) Immunofluorescence staining of α5-pS16 in asynchronous 293A cells. Representative cells at different stages of mitosis can be distinguished by their DAPI staining and are marked with numbers. Scale bar = 20 µm. No phosphosignals were detected in interphase or S16A mutant cells. (**E**) MCF7 cells were arrested at late G2 by RO-3306 treatment (10 µM, 24 h) and released for the indicated amounts of time. Cell lysates were analyzed by Western blot. Induction of H3-pS10 and decline of CDK1-pY15 mark the onset and progression of mitosis. (**F**) left, mating scheme of the indicated strains; right, the *pup2-myc nda3(KM311)* cells were cultured at 30 °C until OD = 0.6~0.8, then shifted to 16 °C and cultured for another 18–24 h to induce mitotic arrest. Cells were lysed for Western blot analysis.

Ser16 of α5 is strictly conserved during evolution (Figure 1A) and is one of the 10 most frequently detected pS/pT sites of human 26S proteasome [18]. Phosphoproteomic studies have documented α5-S16 phosphorylation in various cell types from human, mouse, rat, and in both budding and fission yeast (PhosphoSitePlus, PhosphoGRID, PomBase) [25,26]. Such evolutionary conservation is rarely seen with proteasome phosphosites as the majority of those found in human proteasomes are only present in vertebrates [16]. In most of these studies, α5-S16 phosphorylation was found in mitotic cells, consistent with its location in a SP motif that is typically recognized by CDK/cyclin complexes (Figure 1A). These features suggest that phosphorylation of α5-S16 may have a functional role in regulating mitosis.

To investigate α5-S16 phosphorylation, we generated a phospho-specific antibody against this site (Figure 1B). In human 293T, MCF7 and mouse Neuro2A cells synchronized at M phase by nocodazole (Ndz) treatment, and phosphorylation of endogenous α5-S16 was quite evident from cell extracts as seen by immunoblotting (Figure 1C). Immunofluorescence staining of an asynchronous cell population also showed the presence of α5-pS16 at various stages of mitosis (Figure 1D and Figure S1). To determine the kinetics of α5-S16 phosphorylation, we arrested cells at late G2 by inhibiting CDK1 activity using the potent and specific inhibitor, RO-3306 [27]. After RO-3306 washout, cells soon entered M phase, as evidenced by the induction of H3-pSer10 and a decrease of CDK1-pTyr15, both of which are common mitotic markers (Figure 1E). Notably, α5-S16 phosphorylation closely tracked the profile of H3-pS10, persisted through mitosis, and declined afterwards (Figure 1E). No phospho-signal could be detected in interphase cells or in S16A knock-in cells (Figure 1C–E), and the protein level of α5 did not change with cell cycle (Figure 1C,E). To confirm the evolutionary conservation of α5-S16 phosphorylation, we generated a fission yeast (*Schizosaccharomyces *pombe**) strain with endogenous Pup2 (α5 ortholog) bearing a C-terminal myc tag and crossed it with the nda3-KM311 mutant (Figure 1F). The *nda3* gene encodes the only β-tubulin of *S. pombe*, and the nda3-KM311 mutant is well characterized for its cold sensitivity [28]. This allows for efficient synchronization of yeast cells at the M phase by shifting to 16 °C, a condition known to perturb the microtubule system during mitosis [28,29,30,31]. Indeed, S16 phosphorylation of Pup2-myc was readily seen in the cold-treated mitotic cells (Figure 1F). These pharmacological and genetic manipulations demonstrate that α5-S16 is indeed a mitosis-specific phosphosite that is conserved from yeast to human.

**Figure 2:**
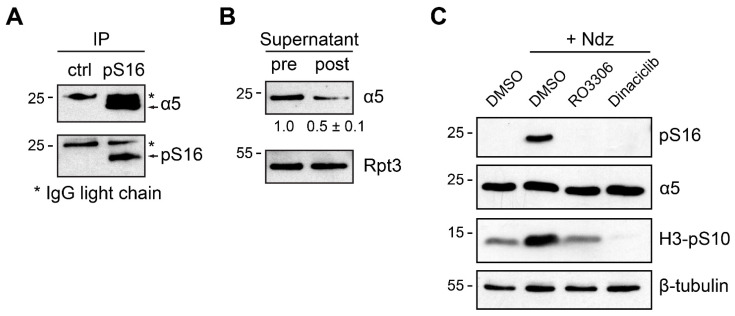
Further characterization of α5-S16 phosphorylation regulated by CDK1. (**A**) Nocodazole-treated 293T cells were lysed for anti-pS16 immunoprecipitation. Arrows point to the endogenous total α5 or phospho-α5 proteins. A normal rabbit IgG was used as control. *, IgG light chain. (**B**) Immunodepletion of S16-phosphorylated α5. 293T cells were first treated with 2.5 mM thymidine for 16 h, then switched to nocodazole treatment (100 ng/mL, 12 h) for a complete synchronization at mitosis. Whole cell lysate (100 µg) was added to an excess amount (10 µg) of anti-pS16 antibody pre-bound to Protein G beads. An identical amount (10 µg) of supernatant was withdrawn from pre- and post-immunodepletion samples and analyzed by anti-α5 Western blot. After depletion of the phosphorylated protein, the amount of remaining total α5 relative to the starting level was quantified from 3 independent experiments. Rpt3 served as loading control, whose level was not affected by anti-pS16 immunoprecipitation. (**C**) 293T cells were treated with nocodazole (100 ng/mL) for 12 h, then incubated with CDK1 inhibitors RO-3306 or dinaciclib (2 µM) for another 4 h before harvest. Total cell extracts were blotted with the indicated antibodies.

Taking advantage of our anti-pS16 antibody that can immunoprecipitate the endogenous protein (Figure 2A), we carried out an immunodepletion assay to gauge the stoichiometry of α5-S16 phosphorylation during mitosis [17]. The results suggest that approximately 50% of α5 was phosphorylated at Ser16 (hence removed by anti-pS16 IP) in nocodazole-synchronized 293T cells (Figure 2B). Such a high level of phosphorylation is surprising but consistent with the high abundance of α5 protein and the easiness of pS16 detection by both straight Western blot and mass spectrometry. In addition, Ser16 phosphorylation could be completely abolished in M phase cells by CDK1 inhibitors such as RO-3306 and dinaciclib (Figure 2C), supporting the idea that phosphorylation of α5-S16 (within a SP motif) depends on and may be directly catalyzed by CDK1/cyclin B, which is highly active in nocodazole-arrested cells.

### 3.2. The α5-S16A Mutation Impaired Cell Proliferation

**Figure 3:**
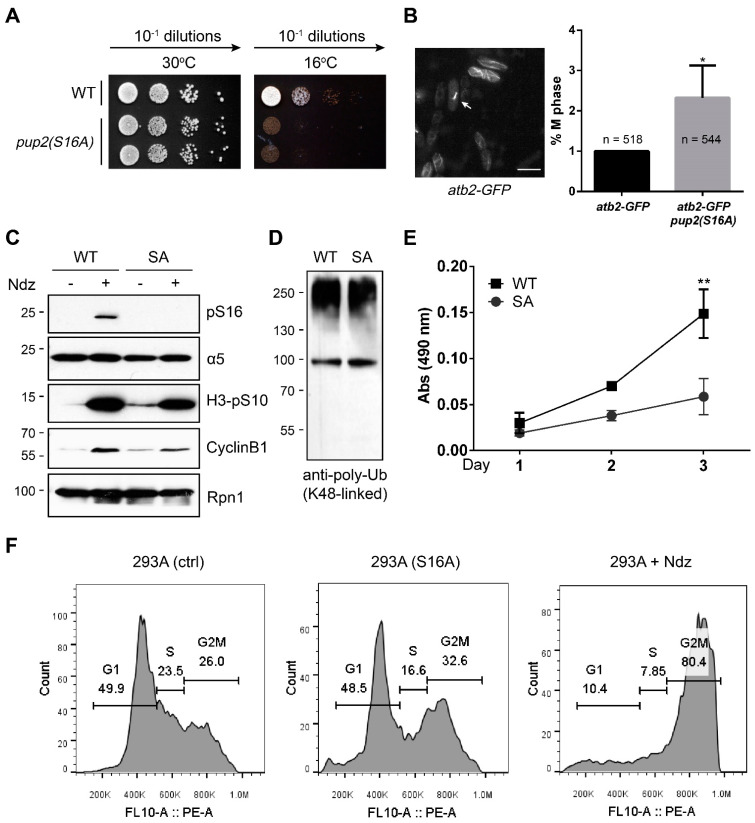
The α5 (S16A) mutation impaired cell proliferation. (**A**) Plate assay with serial-diluted wild-type or *pup2(S16A)* cells. (**B**) Left, a representative confocal image of *atb2-GFP* cells showing the microtubule cytoskeleton. A mitotic cell is indicated by the arrow. Right, *atb2-GFP* and *atb2-GFP pup2(S16A)* cells were grown at 16 °C and imaged. The percentage of mitotic cells from each sample was manually quantified. * *p* < 0.05 (unpaired two-tailed Student’s *t*-test between the indicated groups, from 3 independent experiments). (**C**) Western blot validation of 293A α5-SA knock-in. The 19S proteasome subunit Rpn1 serves as loading control. (**D**) The same lysates of 293A control (WT) and S16A cells used in (C) were probed with a K48-linkage-specific antibody. (**E**) 293A control (WT) and S16A cells were seeded into a 96-well plate at 3000/well in triplicate. Cell proliferation was determined by the MTS assay. ** *p* < 0.01 (two-tailed paired Student’s *t*-test, *n* = 3). (**F**) Flow cytometry analysis of cell cycle distributions of the indicated cells. 293A cells treated with Ndz (100 ng/mL, 16 h) served as a reference.

We then introduced the α5-S16A mutation in yeast and mammalian cells to investigate the biological function of this phosphosite. In *S. pombe*, which has haploid cells, the *pup2(S16A)* mutant showed normal growth and was indistinguishable from the WT cells at 30 °C (Figure 3A). This suggests that the point mutation did not globally perturb proteasome function and cell viability. However, the mutant cells proliferated much more poorly at 16 °C (Figure 3A), exhibiting a clear growth defect. We further crossed the *pup2(S16A)* cells with the *atb2-GFP* strain that expresses a tubulin α2–GFP fusion protein to visualize the mitotic process. Fluorescence imaging of GFP-labeled microtubules/spindles revealed a significant mitotic arrest in the *pup2(S16A)* mutant as compared with WT yeast (Figure 3B).

We also attempted to generate α5-S16A knock-in in human cells using the CRISPR/Cas9 system (Figure S2A). Unlike in yeast, we were unable to obtain homozygous S16A knock-in after extensive trials, probably reflecting the critical importance of this site in mammalian cells. Nonetheless, we did succeed in obtaining heterozygous S16A clones from 293A cells, which were confirmed by genomic DNA sequencing (Figure S2A). We chose 293A (“A” for adherence) over 293T cells because the former is more adherent and easier to manipulate during drug treatments and washes. Both immunoblot (Figure 3C) and immunofluorescence staining (Figure 1D) results demonstrated that α5-S16 phosphorylation was reduced in the S16A cells to a very low level beyond detection. This partial substitution of α5-Ser16 with Ala16 did not alter the total level of α5 protein (Figure 3C), nor did it affect the global poly-ubiquitination status of cellular proteins (Figure 3D). However, it was sufficient to cause a pronounced slowdown of cell proliferation (Figure 3E). Flow cytometry analysis also showed that a higher portion of 293A (S16A) at G2/M as compared to control cells (Figure 3F). On the other hand, heterozygous knock-in of the phospho-mimetic α5-S16D mutation did not interfere with cell proliferation (Figure S2B). These data indicate that α5-S16 phosphorylation is required for optimal proliferation of yeast and mammalian cells.

### 3.3. α5-S16 Phosphorylation Did Not Occur on the Assembled Proteasome Complexes

**Figure 4:**
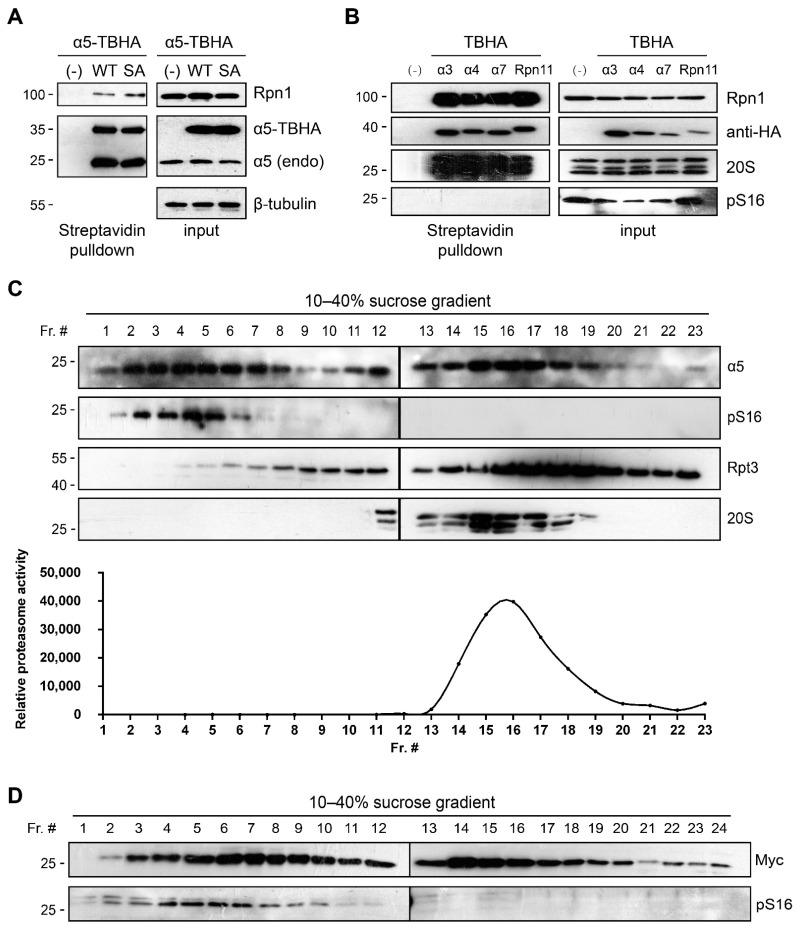
α5-S16 phosphorylation did not occur on the assembled proteasome complexes. (**A**) 293T cells stably expressing the TBHA tag only (−) or the indicated α5-TBHA variants were subjected to streptavidin pulldown assays followed by Western blot. (**B**) 293T cells stably expressing the indicated TBHA fusion proteins were treated with nocodazole (100 ng/mL, 16 h) then lysed for streptavidin pulldown assays as in (**A**). (**C**) 293T cells were treated with nocodazole as in (**B**). Whole cell lysate was fractionated through sucrose gradient ultracentrifugation. Each fraction was analyzed for proteasome complexes by Western blot (top) and proteasome activity using the fluorogenic peptide substrate Suc-LLVY-AMC (bottom). The fraction numbers (Fr. #) are shown. (**D**) The *pup2-myc nda3(KM311)* yeast cells were synchronized at M phase by culture at 16 °C as in Figure 1F. Cells were analyzed by sucrose gradient ultracentrifugation as in (**C**).

We next sought to determine the mechanism by which α5-S16 phosphorylation influences mitosis and cell proliferation. The results above showed that S16 phosphorylation or the S16A mutation did not affect the expression level of α5 or the total level of proteasomes. A series of proteasome pulldown experiments were performed to assess the incorporation status of phosphorylated WT α5 or the α5-S16A mutant in the proteasome complexes. To do so, we individually expressed various proteasome subunits (including α5) with a C-terminal TBHA tag. This tag contains a sequence that can be spontaneously biotinylated (“B” in TBHA for biotinylation) in cells for streptavidin-mediated isolation, as well as an HA tag for immunodetection [17]. Ectopic α5 (WT)-TBHA and α5 (S16A)-TBHA brought down equal amounts of endogenous α5 and the 19S subunit Rpn1 (Figure 4A), indicating stoichiometric assembly of WT and mutant α5 into the 20S and 26S proteasomes. Streptavidin pulldown of the other TBHA-tagged subunits also efficiently captured the whole proteasome from nocodazole-treated cells (Figure 4B). However, we could never detect α5-S16 phosphorylation in any of these pulldown samples (assembled proteasomes), even though the phospho-signal was strong in the whole cell extract (Figure 4B). This result indicates that S16 phosphorylation may only occur on free α5 protein that exists outside the proteasome complex. We therefore fractionated mitotic 293T cell extracts through sucrose gradient centrifugation and examined the distribution of S16-phosphorylated α5. Indeed, the phospho-signal was only found in light fractions containing α5 in the free form or in protein complexes too small to be proteasomes (Figure 4C). The same result was seen with Pup2-myc in *S. pombe* (Figure 4D). Moreover, immunoprecipitation using our anti-pS16 antibody barely brought down any proteasome components from mitotic cells (see Table S4). These findings all point to the fact that α5-S16 phosphorylation exists (and functions) independently from the assembled proteasome. 

**Figure 5:**
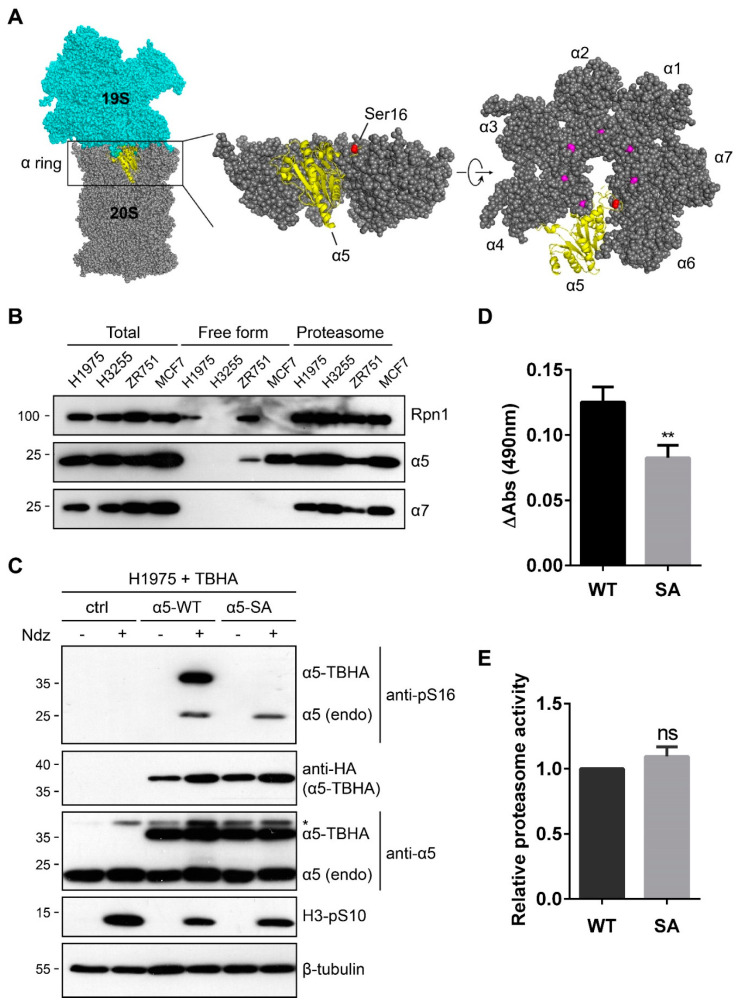
α5-S16 phosphorylation regulated cell proliferation in a proteasome-independent manner. (**A**) Location of α5-Ser16 in the cyro-EM structure of 26S proteasome (PDB: 6msj). α5 is shown as yellow ribbons, while all the other proteasome subunits are shown as spheres. Ser16 is labeled red, and the corresponding Ser residues of other α subunits are shown in magenta. (**B**) Lysates of the indicated cell lines were passed through the same sucrose gradient as in Figure 4. Fractions #3–#7 containing “free” α5 and #15–#19 corresponding to assembled proteasomes were pooled separated and analyzed by Western blot. (**C**) H1975 cells were stably transduced with TBHA only (“ctrl”) or α5-TBHA (WT or S16A). After nocodazole treatment, cell lysates were probed with the indicated antibodies. *, a non-specific band. (**D**) H1975 stable lines expressing α5-TBHA (WT or S16A) were used for MTS assays as in Figure 3E. The increase of Abs (490 nm) from each cell line between day 1 and day 4 is plotted. **, *p* < 0.01 (two-tailed paired Student’s *t*-test, *n* = 3). (**E**) Measurement of proteasome activity from the H1975 stable lines using Suc-LLVY-AMC as substrate. ns, not significant (two-tailed paired Student’s *t*-test, *n* = 3).

The above results may not be unexpected from a structural viewpoint. In the 26S holoenzyme, Ser16 of α5 is close to the central opening of the α ring and buried right at the 19S-20S interface, making it inaccessible to kinases (Figure 5A). However, the high proportion of S16-phosphorylated α5 during mitosis (Figure 2B) indicates that there must be a considerable excess of α5 subunit with regard to the proteasomes in those cells we have examined. This reminded us of several previous studies showing that, in both yeast and human cells, endogenous α5 is expressed at a higher level than any other α subunits of the proteasome [11,12,13,14]. When we analyzed cell extracts of 293T, MCF7, and ZR-75-1 (human breast cancer) cells using sucrose gradient centrifugation, we did find that α5 was abundantly present in both the “free” and “assembled” fractions, whereas the other α subunits (e.g., α7) were primarily found assembled in the proteasome complexes (Figure 5B). We further deduced that the free pool of α5 (and the pS16 signal thereof) would be more sensitive to α5 knockdown and preferentially depleted, as has been suggested for other proteasome subunits [32]. Indeed, in 293T cells treated with an α5-targeting small hairpin RNA (shRNA) that had a moderate knockdown efficiency, the partial decrease of endogenous α5 caused a complete loss of S16 phosphorylation upon nocodazole treatment (Figure S3). On the other hand, a survey of the human Cancer Cell Line Encyclopedia (CCLE) identified H1975 and H3255 cells (two lung cancer lines) with reduced copy numbers of the *PSMA5* gene (encoding α5). Although the protein level of α5 in these cells was only slightly lower than that in the other cell types, we saw a complete lack of free α5 (Figure 5B). Correspondingly, no α5-S16 phosphorylation was observed in H1975 cells, even with nocodazole (Figure 5C, first two lanes). However, stable ectopic expression of α5-TBHA restored the free pool of α5 in H1975 cells, which could be phosphorylated at S16 following nocodazole treatment (Figure 5C, middle lanes). Interestingly, a small fraction of endogenous α5 protein in these cells now also became S16-phosphorylated under the same condition, which must have resulted from their exclusion from the proteasome as being replaced by the exogenous counterpart (α5-TBHA). This also occurred when α5-S16A-TBHA was stably expressed in H1975 cells, although the mutant protein itself remained unphosphorylated (Figure 5C, right lanes). These findings are consistent with the notion that S16A mutation does not interfere with α5 incorporation into the proteasome and indicate that α5-TBHA and endogenous α5 are interchangeable with regard to proteasome assembly. More importantly, this system allowed us to study the gain-of-function effect of α5-S16 phosphorylation in a cell type where it is naturally missing. Compared to WT α5-transduced control, H1975 cells expressing ectopic α5-S16A again proliferated more slowly (Figure 5D), while the overall proteasome activity in these cells remained similar (Figure 5E). α5-pS16 level positively correlated with the rate of cell proliferation. Together, these data revealed a highly conserved phosphosite of a proteasome subunit that appears to function outside the proteasome, which, to our knowledge, is the first example of its kind.

### 3.4. Ser16-Phosphorylated α5 Bound Mitotic Regulators Including PLK1

**Figure 6:**
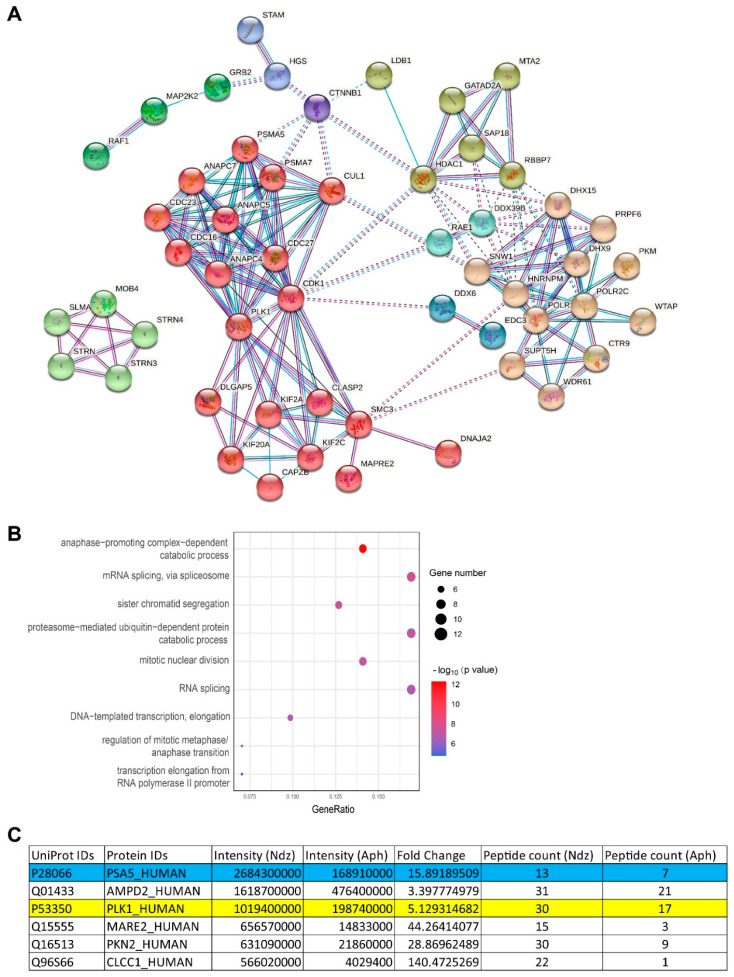
Interactome of S16-phosphorylated α5. 293T cells were treated with aphidicolin (Aph) or nocodazole and lysed for anti-pS16 immunoprecipitation, followed by label-free mass spectrometry analysis. (**A**) Protein–protein interaction (PPI) network of proteins enriched by the anti-pS16 antibody from mitotic cells. (**B**) GO term analysis of proteins shown in (**A**). (**C**) Detailed MS results of the top 5 most abundant and Ndz-enriched proteins (plus α5 itself) from the IP experiment. PLK1 is highlighted.

We reasoned that “free” α5 phosphorylated at Ser16 during mitosis performs important functions by interacting with other cellular factors. Therefore, we immunoprecipitated S16-phosphorylated α5 from cells treated with nocodazole (synchronized in M phase) or with aphidicolin (synchronized in S phase, as control), followed by mass spectrometry analysis. Over 900 proteins were detected from three technical repeats (Table S3). The anti-pS16 antibody captured 16 times more endogenous α5 from the mitotic sample than from the Aph-treated control, demonstrating specific enrichment as predicted. We ranked the proteins according to their intensities, peptide counts, and fold-change between the two samples. A total of 75 proteins were considered to preferentially interact with S16-phosphorylated α5 (Table S4). Protein–protein interaction (PPI) network and gene ontology (GO) term analyses showed that the binding proteins are largely involved in two major functions: mitotic cell cycle and RNA transcription/splicing (Figure 6A,B). Among the top 5 phospho-α5 binders identified (high intensity, high enrichment by nocodazole) was Polo-like kinase 1 (PLK1) (Figure 6C), a master regulator of mitosis. 

**Figure 7:**
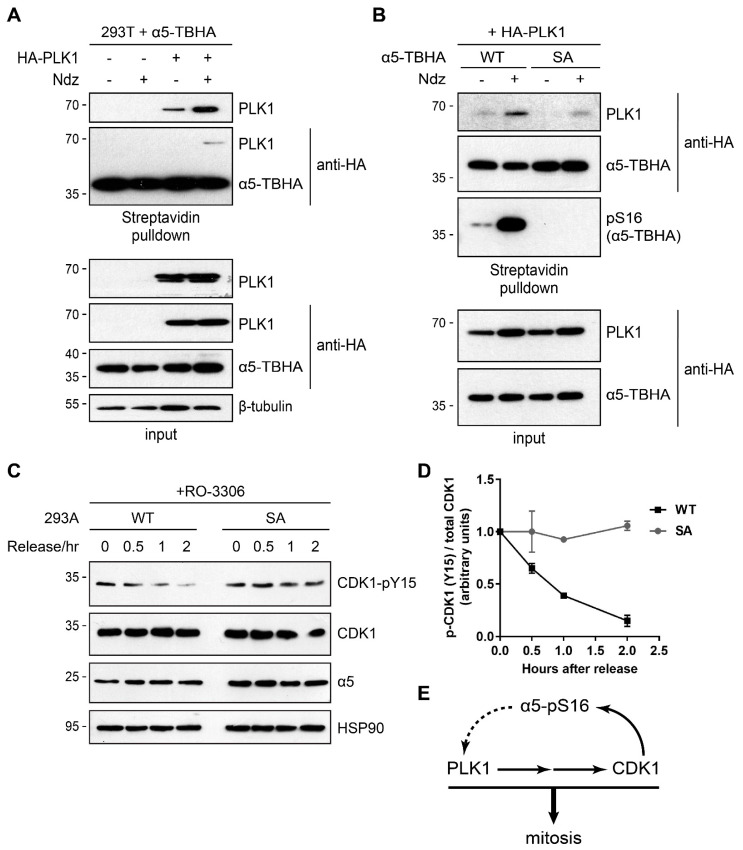
Ser16 phosphorylation facilitates α5-PLK1 binding and mitotic entry. (**A**) 293T cells stably expressing α5-TBHA (WT) were transfected with a vector control (−) or HA-PLK1. Cells were treated with or without Ndz and subjected to streptavidin pulldown and Western blot analysis. (**B**) 293T cells stably expressing α5-TBHA (WT or S16A) were transfected with HA-PLK1. Cells were treated and assayed as in (**A**). (**C**) 293A parental (WT) and S16A knock-in cells were treated with RO-3306 and released as in Figure 1E. Whole cell lysates were probed with the indicated antibodies. HSP90 was used as loading control. (**D**) Quantification of the p-CDK1/total CDK1 ratio in 293A parental (WT) and S16A cells following RO-3306 release (*n* = 3). (**E**) A possible feedforward loop formed by α5, PLK1, and CDK1 drives mitosis.

We transfected HA-PLK1 into cells stably expressing α5 (WT)-TBHA and confirmed their interaction by streptavidin pulldown assay. The result also showed that binding between PLK1 and α5 was much enhanced during mitosis (Figure 7A). Importantly, although α5 (S16A) was also capable of binding with PLK1, their interaction was much weaker and less increased in mitotic cells (Figure 7B). Therefore, Ser16 phosphorylation facilitates α5-PLK1 interaction. PLK1 functions at several steps from mitotic onset to progression [33,34]. In particular, at late G2, CDK1 is inhibited by Wee1- and Myt1-mediated phosphorylation at its Thr14-Tyr15 sites. PLK1 phosphorylates and inactivates Wee1/Myt1 and simultaneously activates the phosphatase Cdc25C that removes the inhibitory phosphorylation of CDK1 [28,33,34]. Therefore, PLK1 is required for full activation of CDK1 upon mitotic entry, which coincides with the appearance of α5-S16 phosphorylation. We examined the CDK1-pY15 level during G2 → M transition, again using the RO-3306 treat/release scheme as in Figure 1E. In 293A α5-S16A knock-in cells, CDK1-Y15 phosphorylation persisted much longer than in control cells after RO-3306 washout (Figure 7C). These results suggest an important role of α5-S16 phosphorylation in promoting mitotic entry and progression, probably by forming a phospho-α5 → PLK1 → CDK1 feedforward loop (Figure 7D).

## 4. Discussion

In this study, we characterized a novel phosphosite of a proteasome subunit, namely, α5-Ser16, and provided indications for its role in regulating mitosis. There are three unique features associated with this phosphorylation event. First, it is extraordinarily conserved through evolution, as far as proteasome phosphosites are concerned. In most cases, this signals functional importance, which we have shown by mutagenesis studies in both yeast and human cells. Second, it occurs on free α5 (monomer or in small protein complexes) as opposed to all the other proteasome phosphosites that have been investigated. Therefore, it is not expected to affect proteasome activity. Third, perhaps as a result of the previous feature, α5-S16 is phosphorylated at an unusually high stoichiometry, which means the majority (if not all) unassembled α5 molecules are phosphorylated during mitosis. On the one hand, this may not be surprising, considering the fact that many mitotically important proteins (e.g., lamin) also become heavily phosphorylated by CDK1. On the other hand, a such high level of phosphorylation is more likely to be involved in structural functions or binding activities rather than switch-like or catalytic roles. 

The above findings led to our prediction that S16-phosphorylated α5 functions outside the proteasome with other proteins to influence mitosis. Among dozens of proteins that were identified by IP/MS to selectively interact with phospho-α5, PLK1 stood out as one of the most abundant interacting proteins with a clearly defined role in mitosis. PLK1 was known to interact with 20S proteasome subunits for nearly two decades, although the biological function thereof has been unclear [26,35]. PLK1 prefers to bind (via its polo box) the phosphorylated motif S[pS/pT]P [36]. Although this motif is absent from α5, the PLK1–α5 interaction does occur. This could mean that PLK1 does not rely on its polo box for phospho-α5 binding, or Ser16 phosphorylation creates a novel PLK1 recognition site on α5, or their interaction might be facilitated by unidentified factor(s). PLK1 has been shown to phosphorylate α3 and α7 but not α5 in vitro [35]. We have found no evidence for α5 to be a substrate of PLK1, consistent with its lack of PLK1 consensus motifs (D/E-X-pS/pT-Φ-X-D/E, where Φ is a hydrophobic residue and X is any amino acid) [37]. 

We speculate that binding of phospho-α5 may positively regulate PLK1 function, which would be consistent with the prolonged CDK1-Y15 phosphorylation observed in S16A cells. Regulation of PLK1 by α5 may occur at several levels, including PLK1 activation, stability, substrate recruitment, or even intracellular localization. These are testable possibilities, despite the fact that difficulty in purifying α5 protein by itself [38] has prevented us from more detailed biochemical assays at the moment. If true, phospho-α5-mediated regulation of PLK1 can promote CDK1 activation, leading to more α5-S16 phosphorylation and formation of a positive feedback loop. On the other hand, in H1975 cells and those alike where α5-S16 phosphorylation does not occur, other mechanisms might have evolved to bypass the requirement of α5-pS16 for rapid cell proliferation. Another interesting question is whether Plo1, the yeast homolog of PLK1, also binds and functions with phosphorylated Pup2. Moreover, we do realize that other factors identified from our IP/MS study (e.g., the APC/C complex) may provide additional explanations to the function of α5-S16 phosphorylation, which certainly warrants further investigation.

We estimated that about half of α5 protein could be phosphorylated at Ser16 during mitosis, which could explain why our S16D heterozygous knock-in cells showed no difference from control cells in proliferation. We screened a good number of CRISPR clones but did not obtain homozygous S16D cells, implying that dephosphorylation of α5-S16 may also be important for cell growth and/or survival. As cells exit mitosis, α5-pS16 signal does decline and becomes undetectable for most of the interphase. Dephosphorylated α5 can re-enter the assembly line to form new proteasome complexes. In this sense, α5-S16 phosphorylation may also serve as a 20S proteasome assembly check point during mitosis. Whether/how S16 phosphorylation blocks α5 association with the other subunits and the biological significance thereof remains to be determined. 

We would like to note that all seven α subunits of eukaryotic 20S proteasomes harbor a conserved N-terminal SP site (α1-S_17_P_18_, α2-S_14_P_15_, α3-S_13_P_14_, α4-S_11_P_12_, α5-S_16_P_17_, α6-S_14_P_15_, and α7-S_16_P_17_ in human, Figure 5A). All these sites have been found to be phosphorylated in human cells, while their phosphorylation information in mouse, rat, and yeast is incomplete with the exception of α5. Given their structural similarity, should the other α subunits exist in the free form, they could be phosphorylated and function in mitosis just like α5. Why evolution has chosen α5 as the proteasome subunit to be produced in excess is an intriguing question. Our study has presented the first example of how an “exo-proteasome” modification of a proteasome subunit participates in cellular functions.

## Data Availability

Raw data of mass spectrometry experiments are available online: http://www.openuaa.com/download/20210407_DJY.zip (accessed on 30 August 2021).

## Supplementary Materials

The following are available online at https://www.mdpi.com/article/10.3390/cells10113075/s1, Figure S1: Immunofluorescence staining of α5-pS16. Asynchronous 293A cells transfected with α5-TBHA were immunostained with anti-pS16 and anti-HA antibodies and imaged with a confocal microscope. Scale bar = 10 µm. The contour of the only mitotic cell in this eye-field is outlined. Note that a neighboring interphase cell transfected with α5-TBHA was negative for pS16. Figure S2: CRISPR/Cas9-mediated knock-in of α5-S16 mutations. (A) Gene editing strategy for knock-in. Thick black bar represents exon 2 (Ex 2) of the *PSMA5* gene where Ser16 is located. LHA and RHA stand for left/right homology arm in the repair donor template. Allele-specific sequencing results of the mutated sites are shown. (B) MTS assay was performed on WT and S16D (heterozygous) cells as in Figure 3E. Figure S3: 293T cells were infected with lentiviruses expressing a control or α5-targeting shRNA. Partial knockdown of endogenous α5 was confirmed by Western blot. Cells were treated with Ndz, and pS16 was analyzed. Table S1: Sequences of primers and oligonucleotides. Table S2: Antibody information. Table S3: All proteins identified from anti-pS16 immunoprecipitation. Table S4: Proteins enriched by anti-pS16 in mitotic cells.
