# Supplementary material for: Conserved Mitotic Phosphorylation of a Proteasome Subunit Regulates Cell Proliferation
Source: Cells. 2021 Nov 8;10(11):3075. doi: 10.3390/cells10113075 (PMC8620773; doi:10.3390/cells10113075)
Supplement: Supplementary file 1 [file cells-10-03075-s001.zip › Supplementary Figures.pdf]

Supplementary Figures

Figure S1

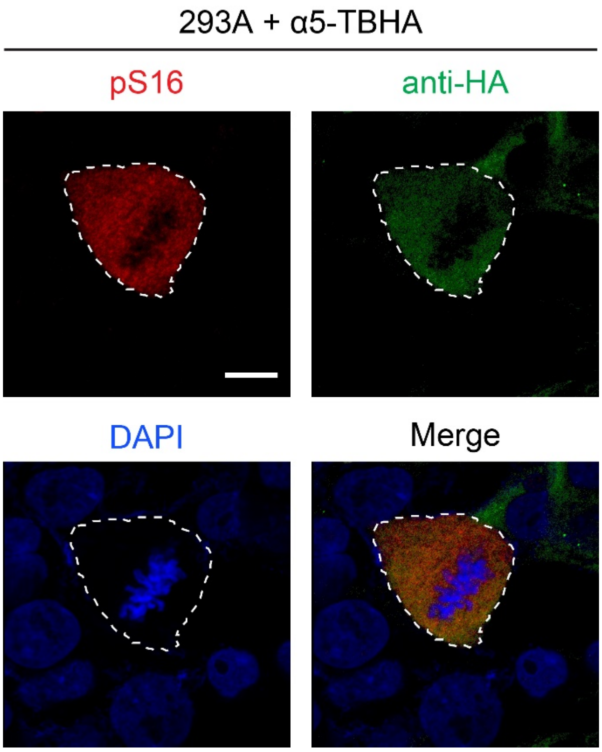

Figure S1: Immunofluorescence staining of α5-pS16. Asynchronous 293A cells transfected with α5-TBHA were immunostained with anti-pS16 and anti-HA antibodies and imaged with a confocal microscope. Scale bar = 10 μm. The contour of the only mitotic cell in this eye-field is outlined. Note that a neighboring interphase cell transfected with α5-TBHA was negative for pS16.

Figure S2

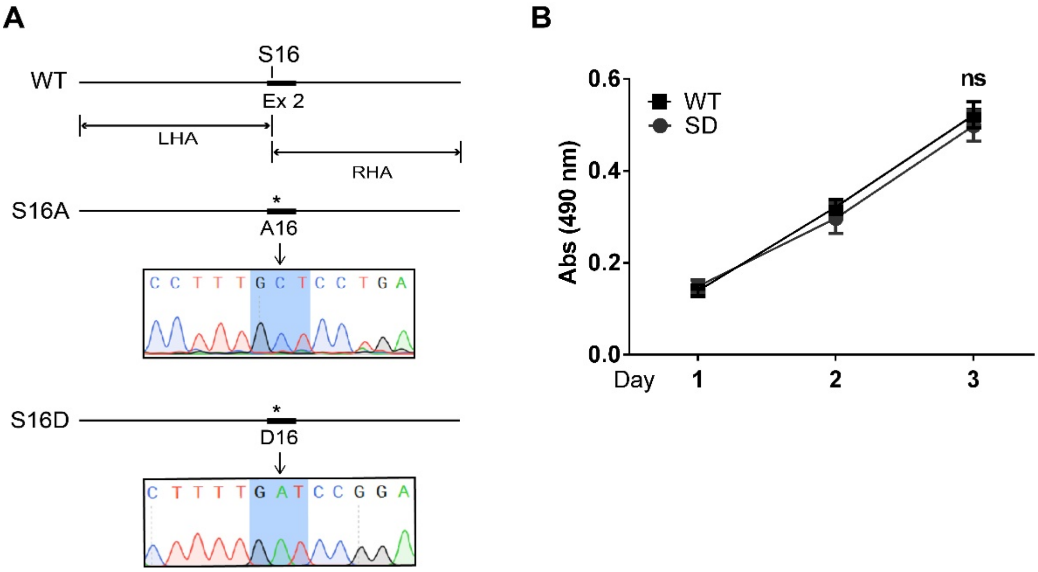

Figure S2: CRISPR/Cas9-mediated knock-in of α5-S16 mutations. **(A)** Gene editing strategy for knock-in. Thick black bar represents exon 2 (Ex 2) of the PSMA5 gene where Ser16 is located. LHA and RHA stand for left/right homology arm in the repair donor template. Allele-specific sequencing results of the mutated sites are shown. **(B)** MTS assay was performed on WT and S16D (heterozygous) cells as in Fig. 3E.

Figure S3

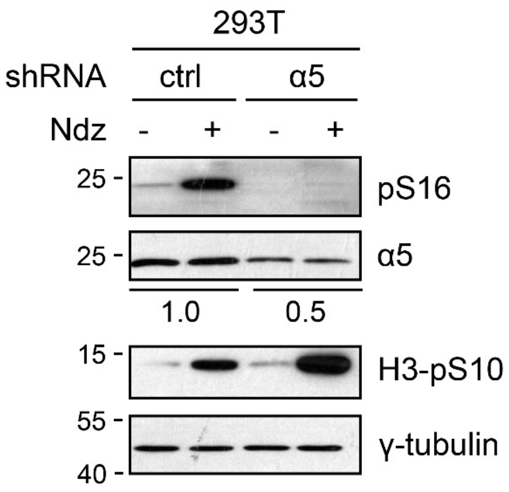

Figure S3: 293T cells were infected with lentiviruses expressing a control or α5-targeting shRNA. Partial knockdown of endogenous α5 was confirmed by western blot. Cells were treated with Ndz and pS16 was analyzed.
